# Encoding of Global Visual Motion in the Avian Pretectum Shifts from a Bias for Temporal-to-Nasal Selectivity to Omnidirectional Excitation across Speeds

**DOI:** 10.1523/ENEURO.0301-24.2024

**Published:** 2024-12-12

**Authors:** Suryadeep Dash, Vikram B. Baliga, Anthony B. Lapsansky, Douglas R. Wylie, Douglas L. Altshuler

**Affiliations:** ^1^Department of Zoology, University of British Columbia, Vancouver, British Columbia V6T 1Z4; ^2^Department of Biological Sciences, University of Alberta, Edmonton, Alberta T6G 2E9, Canada

**Keywords:** directional selectivity, optic flow, pretectum, visual motion, zebra finch

## Abstract

The pretectum of vertebrates contains neurons responsive to global visual motion. These signals are sent to the cerebellum, forming a subcortical pathway for processing optic flow. Global motion neurons exhibit selectivity for both direction and speed, but this is usually assessed by first determining direction preference at intermediate velocity (16–32°/s) and then assessing speed tuning at the preferred direction. A consequence of this approach is that it is unknown if and how direction preference changes with speed. We measured directional selectivity in 114 pretectal neurons from 44 zebra finches (*Taeniopygia guttata*) across spatial and temporal frequencies, corresponding to a speed range of 0.062–1,024°/s. Pretectal neurons were most responsive at 32–64°/s with lower activity as speed increased or decreased. At each speed, we determined if cells were directionally selective, bidirectionally selective, omnidirectionally responsive, or unmodulated. Notably, at 32°/s, 60% of the cells were directionally selective, and 28% were omnidirectionally responsive. In contrast, at 1,024°/s, 20% of the cells were directionally selective, and nearly half of the population was omnidirectionally responsive. Only 15% of the cells were omnidirectionally excited across most speeds. The remaining 85% of the cells had direction tuning that changed with speed. Collectively, these results indicate a shift from a bias for directional tuning at intermediate speeds of global visual motion to a bias for omnidirectional responses at faster speeds. These results suggest a potential role for the pretectum during flight by detecting unexpected drift or potential collisions, depending on the speed of the optic flow signal.

## Significance Statement

During locomotion, images of edges and surfaces in the environment move across the retina, a signal of global visual motion called optic flow. Retinal recipient areas in the accessory optic system and the pretectum are the earliest sites to encode this signal, and the neurons are selective for direction and speed. Previous work suggested that directional selectivity may change across speeds, but this has never been systematically studied. We measured direction preferences from 0.062 to 1,024°/s in the avian pretectum. We found that pretectal global motion neurons are biased for temporal-to-nasal motion at intermediate speeds but biased for omnidirectional responses at faster speeds. These results suggest the pretectum could function to detect both unexpected drift and potential collisions during locomotion.

## Introduction

As an animal moves through the world, the surfaces and edges in the environment appear to move across the retina, generating a global visual signal known as optic flow (Gibson, 1954). Global visual motion is first encoded primarily as a monocular signal in two regions of the midbrain, the accessory optic system (AOS) and the pretectum (Karten et al., 1977; Gamlin and Cohen, 1988; Graf et al., 1988; Soodak and Simpson, 1988). Neurons from these regions exhibit selectivity for direction and speed, but each midbrain site differs in overall population biases. The AOS tends to select for slower speeds (mean typically <10°/s) and has a region of neurons that prefers upward motion, a region that prefers downward motion, and, in some taxa, a region that prefers backward [nasal-to-temporal (NT)] motion (Simpson et al., 1979; Burns and Wallman, 1981; Grasse and Cynader, 1984; Rosenberg and Ariel, 1990). The pretectum, in contrast, has a bias for faster speeds (mean typically > 10°/s) and for forward [temporal-to-nasal (TN)] motion (Collewijn, 1975; Hoffmann and Schoppmann, 1981; Winterson and Brauth, 1985). Both the AOS and pretectum project to the cerebellum and have a role in optokinetic nystagmus (Gioanni et al., 1983, 1984; Simpson et al., 1988a; Lisberger and Sejnowski, 1992; Robinson and Fuchs, 2001). These pathways are also hypothesized to have a role in whole-body stabilization and control (Simpson, 1984; Gutiérrez-Ibáñez et al., 2023).

In addition to direction-selective cells, two other response types have been described in the avian pretectum: bidirectional cells, which respond primarily to opposite directions, and omnidirectional cells, which respond equally well to all directions (Fu et al., 1998; Wylie and Crowder, 2000). A close examination of Wylie and Crowder suggests that direction selectivity could be speed dependent, and a similar argument has been made for the wallaby pretectum (Ibbotson and Mark, 1994). Changes in direction preference were tested across three speeds (6, 15, and 25°/s) in the pretectum of frogs (Fite et al., 1989). Neurons were selective for speed, but did not shift in direction preferences. A broader range of speeds (∼1–240°) was tested for directional responses in area MT of macaques with a moving bar or spot (Rodman and Albright, 1987). Direction preferences were maintained across speeds, but MT neurons have narrower receptive fields compared with global motion neurons in the AOS, pretectum, and macaque medial superior temporal area (Born and Bradley, 2005). Thus, whether directional selectivity is speed dependent has not been systematically tested for neurons responsive to global visual motion across a broad range of speeds.

In previous electrophysiological measurements from neurons in the AOS and pretectum, visual stimulus direction and speed were limited for two reasons. The first was that in the initial studies of these regions, stimulus speeds had an upper limit of ∼100°/s due to technical constraints (Wylie and Frost, 1990). One solution was to shift from dot field stimulus or gratings with a fixed spatial frequency to gratings that sampled the broader spatiotemporal domain (Wylie and Crowder, 2000). By using combinations of gratings that varied in spatial and temporal frequency, stimulus speeds could be tested up to ∼1,000°/s (Smyth et al., 2022). The second limitation was that there were a large number of combinations of directions and speeds. In previous studies, the solution was to fix direction by first determining the preferred direction at one speed and then to test how the cell responded across a range of speeds. Speed tuning has generally been evaluated only in each cell's preferred and, in some cases, antipreferred (AP) directions.

Here, we ask if both stimulus direction and speed are varied, does directional selectivity change across speeds. We performed extracellular recordings from the pretectal nucleus lentiformis mesencephali (LM) in zebra finches (*Taeniopygia guttata*). The avian LM is homologous to the mammalian nucleus of the optic tract (NOT) (Fite, 1985; McKenna and Wallman, 1985). We tested cells in the spatiotemporal domain but used a restricted set of grating stimuli that maximized the range of tested velocities.

## Materials and Methods

The study subjects were 44 adult male zebra finches (*Taeniopygia guttata*). All procedures were approved by the University of British Columbia Animal Care Committee in accordance with the guidelines set by the Canadian Council on Animal Care.

### Surgical and electrophysiological recording procedures

Animals were anesthetized by an intramuscular injection of 65 mg/kg of ketamine and 8 mg/kg of xylazine. Supplemental doses were delivered when the bird exhibited any reflexive movements. Once birds were in the surgical plane, as assessed via the absence of pedal withdraw reflex, they were placed in a custom small bird stereotax (Herb Adams Engineering). The head was secured with ear bars and by clamping the beak on an adjustable arm. The arm was pitched downward 45° relative to the horizontal plane. A subcutaneous injection of 150 µl of 0.9% NaCl solution was made if needed to help maintain hydration and ion balance during surgery. An incision was made to expose the dorsal surface of the skull. A glass pipette with a tip diameter of ∼5 µm was filled with a 2 M NaCl solution and mounted on a motorized micromanipulator. The pipette was moved to the location of the y-sinus. The initial coordinate for the center of the pretectal nucleus lentiformis mesencephali (LM) at this stereotaxic head angle is 2.8 mm anterior and 2.5 mm lateral right to the y-sinus. The right LM was targeted because it receives contralateral projections from the left eye, which was the location of stimulus presentation.

A ground electrode was attached under the skin near the incision position on the head. The electrode and ground were connected via head stage to a single channel amplifier (A-M Systems, model 3000) with a gain of 10,000, and the filters were set wide open. Amplified signals were delivered to an audio monitor (A-M Systems, model 3300) and also to an analog-to-digital data acquisition system (Cambridge Electronic Design, micro1401-3).

**Figure 1.**
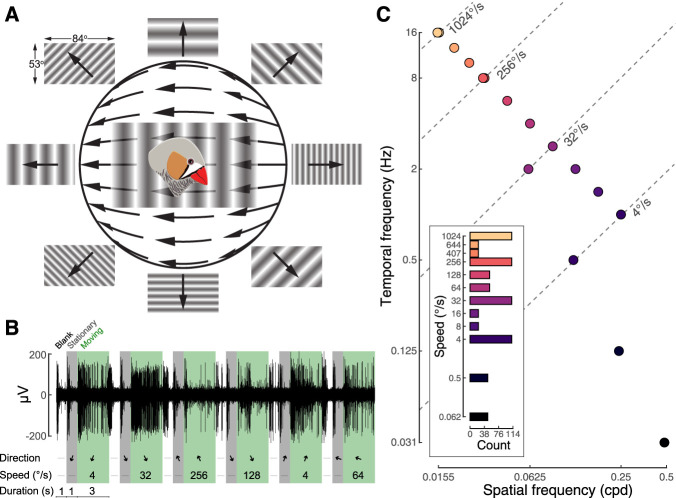
Experimental design for measuring direction preferences of pretectal neurons across a range of stimulus speeds. ***A***, Stimuli were shown on a single screen (84° horizontal × 53° vertical) that was positioned lateral to the bird. Sine-wave gratings were presented in a randomized order that varied in orientation and in spatial and temporal frequency. Different spatial frequencies are depicted here. Each stimulus sweep consisted of 1 s of blank screen, followed by 1 s of stationary stimulus presentation and 3 s of stimulus motion. Orientation was tested in eight directions, 45° apart. The head was pitched downward 45° in the stereotax. Stimulus direction is depicted relative to the orientation of a zebra finch in forward flight, with 0° indicating temporal-to-nasal (TN) motion; 180° indicates nasal-to-temporal, 90° indicates upward, and 270° indicates downward motion. ***B***, A representative recording from a zebra finch LM neuron in response to different speeds and directions (arrows) of visual motion (green) interlaced with periods of a blank screen (white) and a stationary stimulus screen (gray). The arrows indicate the orientation of the stimulus (gray) and both orientation and direction (green). ***C***, Stimulus speed is defined as the ratio of temporal to spatial frequency (dashed diagonal lines). We initially tested 48 cells across a range that spanned from 0.062 to 1,024°/s. Responses to slow speeds were minimal, so we then used a narrower but more densely sampled range from 4 to 1,024°/s. The inset shows the number of cells recorded at each speed. In both experiments, cells were recorded at 4, 32, 256, and 1,024°/s.

The feathers below the left eye were lightly taped to the ear bar to keep the eye open. Pretectal LM neurons in the zebra finch were targeted using a stereotaxic atlas (Nixdorf-Bergweiler and Bischof, 2007). The electrode was lowered while monitoring the recording. We showed global visual motion to the open eye, either through the movement of a large board with complex visual patterns or by placing a video screen in the eye's path while displaying in multiple directions and at multiple speeds. When we encountered a cell that responded to these stimuli, we made an initial assessment as to whether the recorded neuron was pretectal or tectal. The key difference is that pretectal LM neurons respond to moving large-field motion unlike nearby tectal cells, which only respond to small stimuli (Frost et al., 1990). A putative LM neuron was identified when the response was sustained in at least one direction. In this stereotaxic coordinate system, the LM is typically reached at a depth between 5.1 and 7.9 mm. Once a putative LM neuron was identified, the electrode was adjusted to maximize isolation (Fig. 1*B*).

### Stimulus presentation and data acquisition

Two different spatiotemporal stimulus programs were used to study cell responses across a range of visual motion speeds (Fig. 1*C*). In all cases, a stimulus sweep consisted of a blank screen for 1 s, followed by a static black and white sine-wave grating for 1 s, which was followed by that same sine-wave grating in motion for 3 s. The computer that generated the stimulus sent a transitor-transistor-logic pulse with each sweep that was acquired in the data aquisition system and synchronized with the electrophysiological data. A photodiode, attached to the lower corner of the stimulus screen, simultaneously verified the timing of stimulus changes. Eight directions were tested, 45° apart. In our stimulus program, 0° and 180° were aligned with the stereotaxic arm. Based on a high-speed video recording of a zebra finch in flight, we determined that the earth horizontal (nasal-to-temporal) plane for a zebra finch is 20° above a bird's orientation in the stereotax. We define temporal-to-nasal (TN) direction as 0°, the “down” direction as 90°, the nasal-to-temporal direction as 180°, and the “up” direction as 270°. In this coordinate system, the electrophysiological measurements were made at stimulus directions of 20°, 65°, 110°, 155°, 200°, 245°, 290°, and 335°. In the first set of experiments, spatial frequency ranged from 0.0155 to 0.5 cycles per degree (cpd) and temporal frequency ranged from 0.031 to 16 Hz. Six speeds were tested: 0.062, 0.5, 4, 32, 256, and 1,024°/s. These stimuli were programmed using Psychophysics Toolbox 3 in Matlab. For each cell recording, the full set of stimuli was ordered randomly and tested once each, which defined a full stimulus sweep. Up to 10 full stimulus sweeps were performed.

During this first set of experiments, we found that responses at low speeds (<4°/s) were often indistinguishable from the spontaneous rate. We therefore designed a new stimulus program to gain further resolution of response differences at faster speeds. The spatial frequencies ranged from 0.0155 to 0.25 cpd, and the temporal frequencies ranged from 1 to 16 Hz. Up to 10 speeds were tested: 4, 8, 16, 32, 64, 128, 256, 407, 644, and 1,024°/s. All cells in both sets of experiments were tested at 4, 32, 256, and 1,024°/s. We confirmed with high-speed video recording (512 frames per second) that there was no aliasing at any stimulus speed.

Electrophysiological data were acquired, and initial analysis was performed using Spike2 (Cambridge Electronic Design). Raw traces were sorted into single units with isolated spikes (wavemarks) using full-wave templates. The template window width was set to include a full spike, and trigger thresholds were adjusted to exclude noise and capture spikes. Spike-sorted data were exported in Matlab (MathWorks) format for further analysis.

### Cell classification

**Figure 2.**
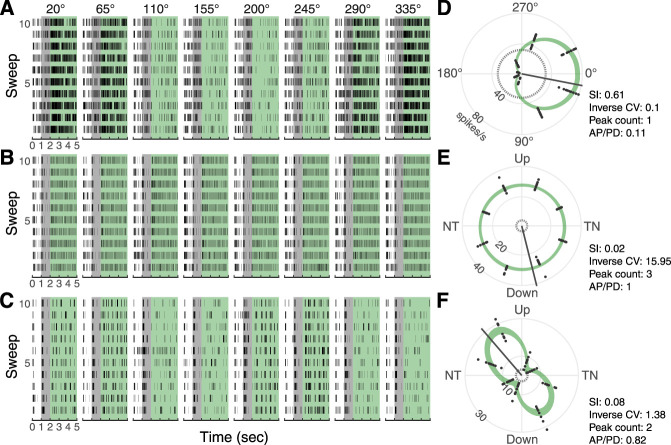
Representative recordings from cells at a stimulus speed of 32°/s that were classified as directional (***A***), omnidirectional (***B***), and bidirectional (***C***). Rasters from 10 sweeps in each direction are aligned. The black vertical lines indicate individual spike timing. Note that directions were randomized within each sweep during recording. The white undershading in the raster indicates the period of the white screen, gray indicates static sine-wave stimulus, and green indicates moving sine-wave stimulus. ***D–F***, Corresponding polar tuning plots are shown for each neuron at 32°/s. The angle indicates stimulus direction, and radius indicates firing rate. The dashed circle indicates the background firing rate (averaged across all static stimulus orientations), and the green polynomial (mean ± SEM) is fit to data for moving stimuli. Neurons were characterized using the inverse coefficient of variation (CV), sensitivity index (SI), ratio of firing rate in the antipreferred direction to that in the preferred direction (AP/PD), and peak count.

We generated a diagnostic analysis for each cell’s responses, which included raster plots, peristimulus time histograms, and polar tuning plots (Fig. 2). This initial analysis revealed transient activity as the stimulus changed from blank screen to stationary stimulus to moving stimulus and back to blank screen. The transient responses lasted up to 200 ms. We calculated the spontaneous firing rate for each cell as the average response during the period of 500–1,000 ms when all of the stationary stimulus patterns were displayed. We next calculated the average response to moving stimuli for each sweep at a given speed and direction over the motion epoch. At this stage, some cells were excluded from further analysis because they did not meet the criteria for being selective for global visual motion. The inclusion criteria required that the cell exhibited the following for at least one speed: (1) a sustained response to at least one stimulus condition across sweeps and (2) a response to at least one direction with a firing rate greater than or equal to 5 spikes/s above the spontaneous firing rate. Following diagnostic checks, we had a total sample of 114 neurons and a total sample of 924 cell responses across speeds (Fig. 1*C*, inset).

We next generated polar tuning plots and fitted a natural cubic spline to these data, with 7 or 8 df. The polar tuning plots revealed that the responses could be categorized based on the shape of the curves. A curve with a single prominent peak illustrates a “directional” preference. Some curves had two peaks, typically 180° apart, and therefore represent “bidirectional” activity. We also noticed that some cells were responsive to all directions of motion, which we termed “omnidirectional.” Finally, some cells that were active at one or more speeds were unresponsive to any direction of global visual motion at other speeds. We term this lack of response as “unmodulated.”

To aid in the classification of the 924 cell responses at each stimulus speed, we calculated several response properties. For all of these response properties, we subtracted the mean spontaneous rate from the firing rate in response to a visual stimulus. The preferred direction of each cell at each speed was calculated using the vector sum:

$$\text{Preferred direction}=\tan^{-1}\;\left(\frac{\sum_{n}\;(FR_{n}\;*\;\sin\theta_{n})}{\sum_{n}\;(FR_{n}\;*\;\cos\theta_{n})}\right),$$

where FR_*n*_ is the average firing rate in response to direction n for all eight directions of motion presented (in radians).

The tuning properties of LM neurons were characterized using four other parameters. The width of the direction tuning curve was calculated using the sensitivity index (SI), which is defined as the normalized length of the mean response vector (Vogels and Orban, 1994):

$$\text{SI}=\frac{\sqrt{{\left(FR_{n}\;*\;\sin\theta_{n}\right)}^{2}+{\left(FR_{n}\;*\;\cos\theta_{n}\right)}^{2}}}{\sum_{n}\;FR_{n}},$$

The SI ranges from 0 to 1, with an SI of 0 indicating a neuron responding equally to all measured directions of motion and an SI of 1 indicating that a neuron responds only to a single motion direction. Another measure of the strength of direction tuning is the ratio of the firing rate in the antipreferred direction to the firing rate in the preferred direction (AP/PD). The AP is opposite (180° away) from the PD. We also calculated the ratio of the mean of the firing rate across all directions to the standard deviation of the average firing rates in each direction. This measure is higher for cells that are responsive to many directions and is the inverse of the coefficient of variation (inverse CV). Finally, we implemented the findpeaks function in pracma (Borchers, 2023) to determine the peak count.

Cell responses at each speed were classified based on the shape of the turning curves using a machine learning approach. To establish a training data set, we focused on classifying the response of each cell at the speed at which the cell's response was most active (i.e., the speed at which the response in the preferred direction was greatest vs the cell's spontaneous rate). In these “most active” conditions, all 114 cells exhibited activity above the spontaneous firing rate and could be manually classified into one of three categories: bidirectional, directional, or omnidirectional. Our manual classifications generally relied on assessing the overall shape of the tuning curve but were also aided by whether SI was >0.2, which was generally indicative of directional classification. To ensure the training data set was not systematically biased by the most active responses, we manually classified an additional 100 modulated responses, choosing cells and speeds randomly. In an initial approach, we had included cell responses that were “unmodulated” as a potential category but found doing so resulted in poor performance (high misclassification rate). This was likely due to unmodulated responses having tuning curves that could be similar in shape to those of directional, omnidirectional, or bidirectional responses, albeit at an overall lower spike rate. We therefore elected to perform two stages of analysis: (1) categorize all responses based on the shape of the tuning curve using machine learning, and (2) reclassify some responses as unmodulated based on additional criteria.

**Figure 3.**
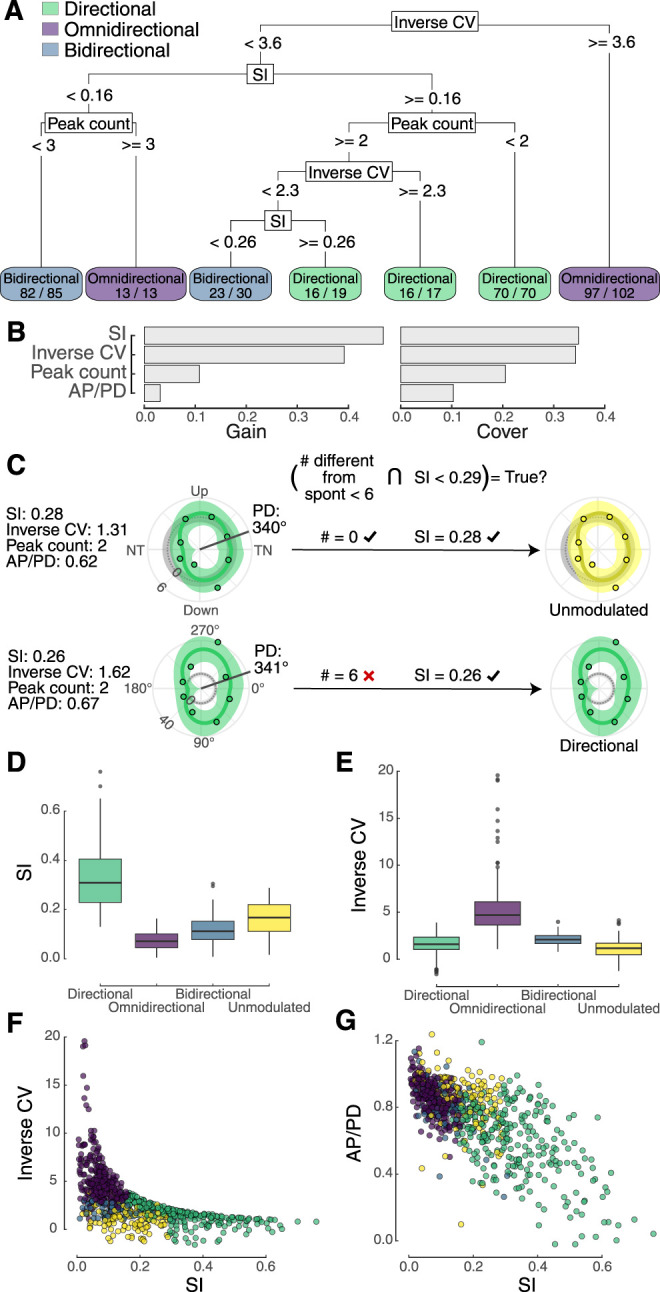
Pretectal neurons were classified in two stages using several measures of neural activity. In the first stage, cells were classified as directional, bidirectional, or omnidirectional based on selectivity index (SI), inverse of the coefficient of variation (CV), ratio of firing rate in the antipreferred to preferred direction (AP/PD), and peak count. ***A***, A representative example of a decision tree used by XGBoost to classify cells in the first stage is shown. This example has high accuracy for the training data for which it was supplied, based on the success ratios shown at the bottom. The XGBoost model was built from >2,500 decision trees. ***B***, The relative contribution (gain) and relative number of observations (cover) in the consensus model reveal that SI and inverse CV were the most informative, whereas peak count and AP/PD provide refinement. ***C***, In the second stage, cells can be reclassified as unmodulated if two conditions were true: (1) fewer than six directions had mean firing rates that were significantly different from the spontaneous rate, and (2) SI ≤ 0.29. This step is illustrated for two cells with similar preferred directions (PD) and similar activity characteristics. The top cell is reclassified as unmodulated because its activity in most directions is indistinguishable from the spontaneous firing rate (gray circle) and its SI = 0.29. The bottom cell is directional even though its SI is lower because its activity in six directions is above the spontaneous rate. It is not bidirectional because its SI is >0.2. The mean spontaneous rate has been subtracted from all data and is therefore shown at 0 spikes/s (gray). ***D***, A boxplot of SI values illustrates that this measurement was informative for identifying directional responses. ***E***, In contrast, inverse CV was informative in identifying omnidirectional responses. Bivariate plots of inverse CV (***F***) and AP/PD (***G***) versus SI provide graphical representations of how cells segregate after two stages of classification. Additional detail is provided in Extended Data Figure 3-1.

In the first stage of classification, we used extreme gradient boosting via XGBoost (Chen and Guestrin, 2016). Boosting is an ensemble extension of random forest modeling: decision trees are fit to train data sequentially to improve upon preceding outcomes. An example of a decision tree that could have been used during boosting is shown in Figure 3*A*. The target variable for the model was the manually classified responses from the training data set. The features included SI, inverse CV, AP/PD, and peak count. To improve generalizability, we performed repeated *k*-fold cross-validation, with five repeats and with *k* = 5. Additional details of the tuning grid, including boosting rounds, eta, gamma, and subsampling, are available in our code repository (Baliga et al., 2024). The best-tuned model was determined and found to have 100% accuracy on the training data set (chi-square: 426, *p* < 0.001, Table 1) as well as on several test data sets. The parameters of SI and inverse CV were the most informative for the model, both in terms of their relative contributions (gain) and relative number of observations (cover; Fig. 3*B*). The parameters of peak count and AP/PD provided further refinement. This model was subsequently used to predict the categorization of all 924 cellular responses (Fig. 3*D–G*, Extended Data Fig. 3-1). Response classifications were thereafter spot-checked and, in all cases, found to agree with manual classification.

**Table 1.** Summary of formal hypothesis testing conducted

Data structure	Type of test	Power
Categorical (cell shape categories)	Chi-square	df = 426, *p* < 0.001

df, degrees of freedom.

10.1523/ENEURO.0301-24.2024.f3-1 Figure 3-1 Pretectal neurons were classified in two stages using several measures of neural activity. In the first stage, cells were classified as directional, bidirectional, or omnidirectional based on selectivity index (SI), inverse of the coefficient of variation (CV), ratio of firing rate in the anti-preferred to preferred direction (AP/PD), and peak count. A) A representative example of a decision tree used by XGBoost to classify cells in the first stage is shown. This example has high accuracy for the training data for which it was supplied, based on the success ratios shown at the bottom. The XGBoost model was built from > 2500 decision trees. B) The relative contribution (gain) and relative number of observations (cover) in the consensus model reveals that SI and inverse CV were the most informative, whereas peak count and AP/PD provide refinement. C) In the second stage, cells can be reclassified as unmodulated if two conditions were true: i) fewer than six directions had mean firing rates that were significantly different from the spontaneous rate, and ii) SI ≤ 0.29. This step is illustrated for two cells with similar preferred directions (PD) and similar activity characteristics. The upper cell is reclassified as unmodulated because its activity in most directions is indistinguishable from spontaneous firing rate (grey circle) and its SI = 0.29. The lower cell is directional even though its SI is lower because its activity in six directions is above the spontaneous rate. It is not bidirectional because its SI is > ~0.2. Mean spontaneous rate has been subtracted from all data and is therefore shown at 0 spikes/s (grey). D) A matrix of plots for SI, Inverse CV, Peak count, AP/PD, and classification, colored by classification. Each row and column is labeled with one of the five variables. The plots on the unity diagonal show density histograms. Cells below the diagonal are bivariate plots. Cells above the diagonal provide overall correlations and classification-specific correlations, or (for the rightmost column) box plots. Download Figure 3-1, TIF file.

In Stage 2, we reclassified some responses as unmodulated (Fig. 3*C*). A cell's response can be considered unmodulated if it is not sufficiently distinguishable from the cell's spontaneous rate. We applied a rule wherein two conditions were checked: (1) whether a cellular response was not statistically different from the spontaneous rate in more than six directions and (2) if SI < 0.29. If both conditions were true, the cell response was reclassified as unmodulated.

### Data analysis

To facilitate comparisons of how LM direction responses changed with speed, we normalized firing rates within cells and across speeds. Data within each cell were normalized to the absolute value of the maximum response among all speeds and directions. This defines *R*_n_, the “normalized directional response,” as ranging from −1 (maximal possible suppression) to +1 (maximum response recorded). Because the spontaneous firing rate had already been subtracted prior to this normalization, the spontaneous rate was defined as 0 for the normalized response.

A response feature that became apparent during diagnostic analysis is that the duration of the responses also varied with speeds. To facilitate analysis of responses through time, we separately normalized firing rates within cells and across time bins to define the “normalized temporal response.” At each speed and each direction, the response to the motion epoch was divided into 10 ms bins. The spontaneous rate of the cells was subtracted from each bin. The bins were normalized to the absolute value of the maximum response across all such bins for a given cell. As above, this led to a given cell's maximum response being defined as 1, its spontaneous rate being defined as 0, and its maximum possible level of suppression being defined as −1.

Uncertainty bands in figures are 95% confidence intervals, which are used in many cases for comparisons among fitted curves. Statistical trends in response properties with stimulus speed were assessed by comparing goodness of fit via Akaike information criterion (AIC) among candidate generalized additive models (GAM). To assess speed tuning, we compared the following three models:

$$R_{n}∼\;(1\;|\;\mathrm{cell}),$$

$$R_{n}∼\;s(\log_{2}(\mathrm{speed})),$$

$$R_{n}\;∼\;s(\log_{2}(\mathrm{speed}))+(1\;|\;\mathrm{cell}),$$

where *s* is the GAM smoothing function.

In a separate set of analyses, we tested how the time to peak activity (*time*_p_) changes with stimulus speed. Here, the responses over time were compared via AIC using the following five GAM models:

$$\log_{2}\;(\mathrm{tim}e_{p})∼\;(1\;|\;\mathrm{cell}),$$

$$\log_{2}\;(\mathrm{tim}e_{p})∼\;s(\log_{2}(\mathrm{speed})),$$

$$\log_{2}\;(\mathrm{tim}e_{p})\;∼\;s(\log_{2}(\mathrm{speed}))+(1\;|\;\mathrm{cell}),$$

$$\log_{2}\;(\mathrm{tim}e_{p})∼\;\mathrm{shape}+\;s(\log_{2}(\mathrm{speed})\;*\;\mathrm{shape}),$$

$$\log_{2}\;(\mathrm{tim}e_{p})\;∼\;\mathrm{shape}+\;s(\log_{2}(\mathrm{speed})\;*\;\mathrm{shape})+(1\;|\;\mathrm{cell}),$$

where shape is a discrete variable that can have one of three states: directional, bidirectional, or omnidirectional. Unmodulated cells were excluded. Separate model fitting was performed for two data sets: one where data were averaged across all eight directions and one where only data from the direction closest to the preferred direction were used.

We also tested how the magnitude of peak activity within each phase (*activity*_p_) changes with stimulus speed. Here, the responses over time were compared via AIC using the following five GAM models:

$$\mathrm{activit}y_{p}\;∼\;(1\;|\;\mathrm{cell}),$$

$$\mathrm{activit}y_{p}\;∼\;\mathrm{phase}+\;s(\log_{2}(\mathrm{speed})\;*\;\mathrm{phase}),$$

$$\mathrm{activit}y_{p}\;∼\;\mathrm{phase}+\;s(\log_{2}(\mathrm{speed})\;*\;\mathrm{phase})+\;(1\;|\;\mathrm{cell}),$$

where phase is a discrete variable that can have one of three states: initial transient, transitional, or steady state. Again, separate model fitting was performed for two data sets: one where data were averaged across all eight directions and one where only data from the direction closest to the preferred direction were used.

### Code accessibility

The spike-sorted electrophysiological data and analysis code are available via Figshare (Baliga et al., 2024).

## Results

**Figure 4.**
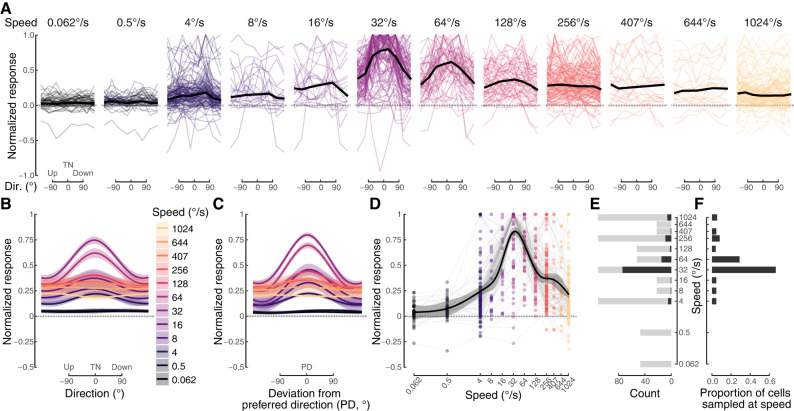
Pretectal neurons are most responsive to stimulus speed of 32°/s, and at this speed, many cells are tuned to temporal-to-nasal (TN) motion (direction, 0°). ***A***, Each thin line shows the normalized response across directions for a single cell at a single stimulus speed. The thick black line is the median response across directions for all cells tested at that speed. Speeds are indicated by panel headings and color. ***B***, The mean (±SEM) of all cell responses across directions is shown for each stimulus speed. ***C***, A similar plot as on the left, but each cell's maximum response has been aligned to 0°. ***D***, The dots show the maximum normalized response of each cell at each measured speed, regardless of direction. A cell is connected by gray lines. The thick black line is the mean (±SEM) speed tuning curve, independent of directional selectivity. ***E***, The sample size at each stimulus speed is shown by the light gray bars. The black indicates the number of cells that were maximally responsive at each speed. ***F***, Dividing the black count by the light gray count provides the proportion of cells that were maximally responsive at each speed.

Pretectal LM neurons were most responsive at intermediate speeds (32–64°) and declined at slower and faster speeds (Fig. 4). The normalized directional responses are shown grouped by speed in Figure 4*A*. At intermediate speeds, the directional tuning curves tended to be relatively sharp and centered at 0°, which corresponds to temporal-to-nasal (TN) motion. At slower speeds, especially below 4°/s, the neuron responses were considerably reduced. At faster speeds (>64°), the cells remained active, but the tuning curves were flatter indicating a shift toward omnidirectional responses. Suppression, which is indicated by negative values in the normalized response, was relatively infrequent.

The mean responses at each speed with the 95% confidence intervals are shown in Figure 4*B*. The TN population bias is strongest at 32 and 64°/s but also present at 16 and 128°/s. At all speeds >4°/s, the population shows responses to global visual motion, and at speeds >128°/s, the population response is relatively uniform across directions. We further examined these differences by aligning all tuning curves at each cell's preferred direction at each speed (Fig. 4*C*). Because of the consistently strong bias for TN motion at intermediate speeds and the more uniform responses at faster speeds, this display of speed-specific tuning responses was largely unchanged. The widths of the directional tuning curves are relatively broad, typically spanning more than ±45° of the preferred direction.

To generate a population speed tuning curve (Fig. 4*D*), we plotted each cell's maximum normalized directional responses at each speed. The best-fitting GAM model (Table 2) indicated that cells generally achieved their highest normalized responses ∼32°/s and that cell identity did not have a meaningful effect on the overall relationship between normalized response and log_2_ of speed. The speed at which each cell reached its measured maximum response is shown in black in Figure 4*E*. Because sample size varied due to two different experimental protocols (Fig. 1*C*), we normalized these data to the sample size at each speed (Fig. 4*F*). The majority of zebra finch LM neurons have their highest responses to global visual motion at 32°/s.

**Table 2.** Goodness of fit metrics for models fit to explain the normalized directional response $\left(R_{n}\right)$

Formula	*R* ^2^	Root mean squared error	Sigma	AIC
$R_{n}∼\;(1\;|\;\text{cell})$	0	0.36	0.36	756.23
$R_{n}∼\;s(\log_{2}(\text{speed}))$	**0.52**	**0**.**25**	**0**.**25**	**135**.**58**
$R_{n}\;∼\;s(\log_{2}(\text{speed}))+(1\;|\;\text{cell})$	0.52	0.25	0.25	137.26

Three GAM models were fit, with potential explanatory variables including log_2_ of stimulus speed (speed) and cell identity (cell; as a random effect). Sigma column denotes the residual standard deviation. The best-fitting model, determined by lowest AIC score, is in bold.

**Figure 5.**
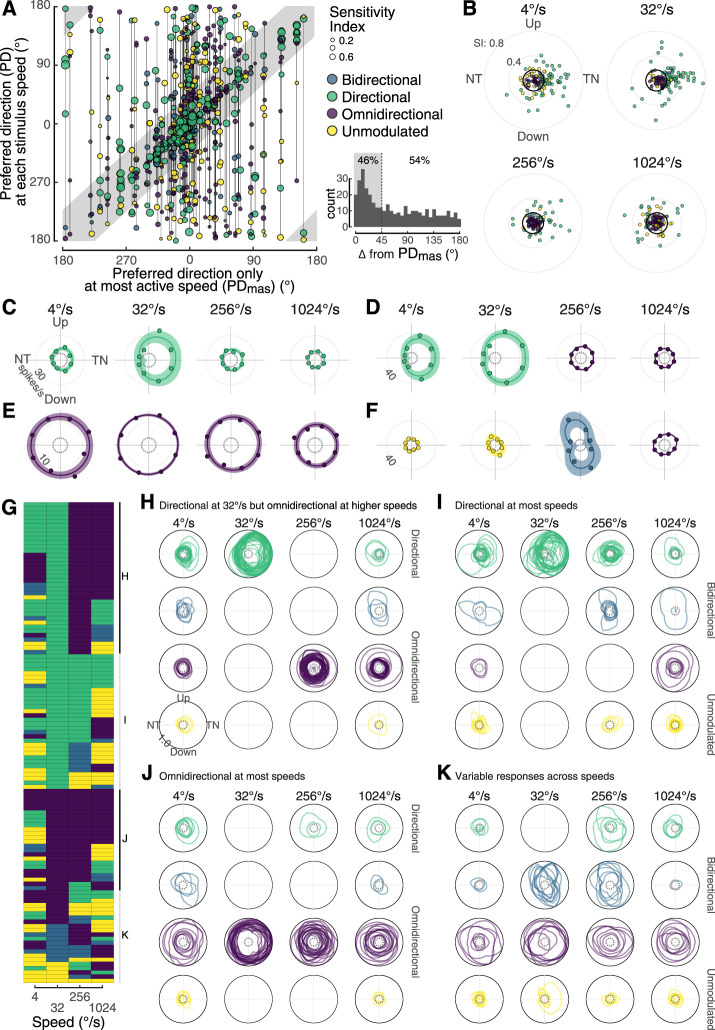
The population of pretectal neurons shifts from a bias for directional tuning at intermediate speeds to a bias for omnidirectional responses at faster speeds. ***A***, Scatter plot of preferred directions for all 924 responses. Each vertical line connects a single cell with its position on the *x*-axis determined by its preferred direction at the speed at which it was most responsive. The *y*-axis shows its preferred directions at all speeds at which it was tested. The size of the circle corresponds to SI, and the color indicates classification. The bounds of the gray undershading are offset by ±22.5° from the line of equivalence. ***B***, All cells were recorded at four speeds: 4, 32, 256, and 1,024°/s. Each response is depicted in a polar plot. The angular position of each point represents a cell's direction preference, and the radial position represents SI. The black circles represent an SI of 0.17, above which cells tended to be directional. Example tuning curves are provided for cells that were (***C***) directional at all four speeds, (***D***) shifted from directional to omnidirectional, (***E***) were omnidirectional at all four speeds, and (***F***) were bidirectional at one speed. The spontaneous rate has been subtracted from the mean response in each direction. ***G***, A tile plot of all cell classifications at each of the four common speeds. Each row is a single cell and row order is determined by classification at 32°/s. This ordering of the tile plot suggests that the cells can be grouped into four categories: directional at 32°/s but omnidirectional at higher speeds (***H***), directional at most speeds (***I***), omnidirectional at most speeds (***J***), and variable across speeds (***K***). Whereas the polar plots in ***C–F*** are shown with the radius in spikes/s, the radii of the plots in ***H–K*** are normalized to the maximum firing rate of each cell.

We next asked if preferred directions changed across stimulus speeds. For each cell, we plotted the preferred direction at each measured speed against the preferred direction at each most active speed, depicting its speed-specific classification and SI (Fig. 5*A*). If each cell's preferred direction had been maintained within 45° across speeds, all of the dots would have fallen within the gray region. Of the cells that were sampled at all four common speeds (4, 32, 256, 1,024°/s), nearly half (46%) of the cellular responses fall within this zone, and the other half (54%) are outside of it (Fig. 5*A*, inset). LM neurons tend to be directional and prefer TN motion, but these characteristics are most apparent at speeds of 32°/s and to a lesser extent at 4°/s (Fig. 5*B*). Relatively few of the cells were directional at faster speeds, and there was no overall bias for TN motion among those that are.

Because it is clear that direction tuning changed across speeds, we also analyzed how cell classification changes. Examples of cells that maintained directional (Fig. 5*C*) and omnidirectional (Fig. 5*E*) classification across the four common speeds illustrate that response strengths also varied across speeds. A commonly observed pattern was for cells that were directional at intermediate speeds to shift to omnidirectional at faster speeds (Fig. 5*D*). Bidirectional cells were rare, and none maintained this classification across speeds. An example of a cell that was bidirectional at only 256°/s is shown in Figure 5*F*. The cell classification for all cells at the four speeds that were commonly tested is shown in a tile plot, with cells are ordered based on classification at 32°/s (Fig. 5*G*). This ordering suggests that responses across speeds can be grouped into four categories. Thirty-six out of 114 neurons were directionally tuned (green) at 32°/s but shifted to being omnidirectional at 256°/s. The majority remained omnidirectional at 1,024°/s. The tuning curves for the 36 cells in this category are shown in Figure 5*H*. The next category consists of 32 neurons that are primarily directional. All of these cells were directionally tuned (green) at 32°/s. Most of them were also directionally selective at either 4 or 256°/s, but only four of these were directionally tuned across all directions (Fig. 5*I*). The third category is for the 24 LM neurons that were omnidirectional at most speeds (panel J). The last category is composed of 22 cells with variable responses, including cells that were bidirectional at 32°/s. Note that the polar plots in panels C–F are shown with the radius in spikes/s and the radii of the plots in panels H–K are normalized to the maximum firing rate of each cell.

**Figure 6.**
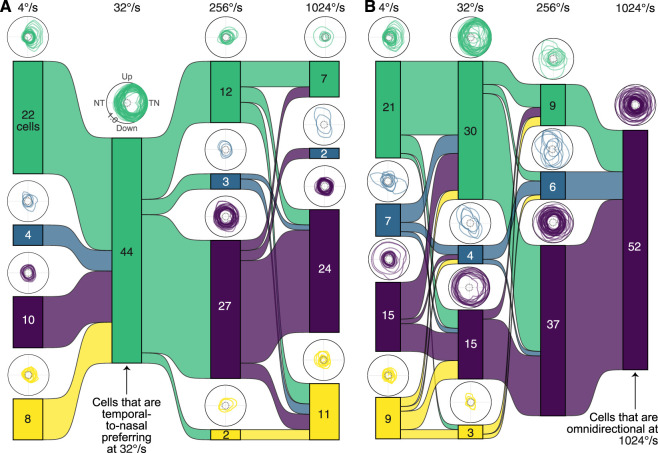
Individual LM neurons differ in their directional tuning across speeds. ***A***, All 44 of the LM neurons that are temporal-to-nasal preferring at 32°/s are shown. The Sankey plot illustrates how the classification of these 44 cells may change at 4°/s, 256°/s, and 1,024°/s. Polar plots above each block contain the normalized directional tuning curves for all cells within the block. ***B***, An analogous Sankey plot is shown for the 52 LM neurons that were omnidirectionally responsive at 1,024°/s.

We have previously demonstrated that the majority of zebra finch LM neurons prefer TN motion at intermediate speeds (Gaede et al., 2017; Smyth et al., 2022), as is the case for most vertebrates. In the current study, 44 of the 114 neurons were both directional and TN tuned at 32°/s. To examine how these cells change in direction tuning across speeds, we made a Sankey diagram (Fig. 6*A*). Only seven of these cells were directional at 1,024°/s, and of these, only three of them remained TN selective. The most common pattern was for cells to become omnidirectional at faster speeds. The tendency is also apparent from a second Sankey diagram, which is composed of all 52 cells that were omnidirectional at 1,024°/s (Fig. 6*B*). The majority of these (30 out of 52) were directional at 32°/s.

**Figure 7.**
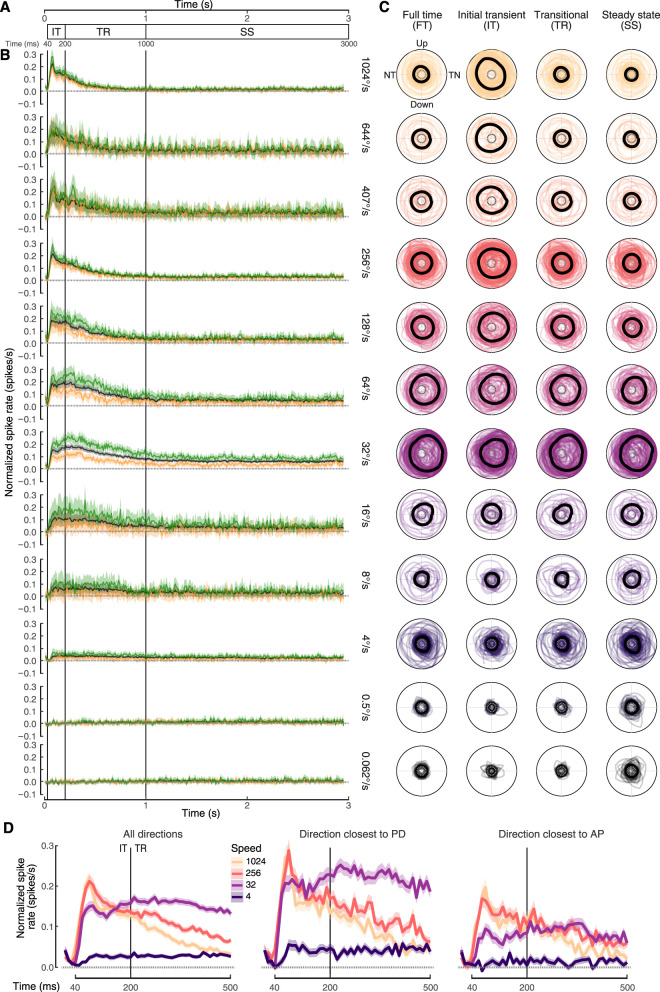
Responses in pretectal neurons are maintained throughout stimulus presentation at intermediate stimulus speeds but are transient and rapid at faster speeds. ***A***, A schematic of responses (spikes/s) over 3 s, which is the motion epoch of the stimulus [full time (FT)]. The response can be divided into the initial transient (IT, 40–200 ms), transitional (TR, 200–1,000 ms), and steady-state (SS, 1,000–3,000 ms) phases. ***B***, Average of normalized spike rate (±SEM) during the entire motion epoch for all cells recorded at each speed. The black line indicates the average response across all directions. The green line indicates the averages of the responses of each cell at the recorded direction closest to that cell's preferred direction. The average response in the opposite recorded direction (180° away) is shown in yellow. ***C***, Polar plots of normalized responses for the FT, IT, TR, and SS phases. Individual cell responses are normalized within each column (phase) by scaling to whichever speed/direction combination had the highest activity. The thick black line is the median response across directions for all cells within each polar plot. ***D***, Averages of spike rate (±SEM) during the first 0.5 s are shown for all directions, the direction closest to the preferred direction (PD), and 180° opposite to this [antipreferred (AP)]. For all panels, the spontaneous rate after normalization is 0 spikes/s and is shown as dotted gray lines.

A previous study of LM responses to large-field moving stimuli demonstrated that the cells have a strong initial transient followed by a sustained steady-state (SS) response (Smyth et al., 2022). This prior result next led us to ask if there are relationships between stimulus speed and cell response dynamics. We divided the response of each epoch of moving stimuli into an initial transient (IT) phase (40–200 ms), a transitional phase (TR, 200–1,000), and a steady-state phase (1,000–3,000 ms; Fig. 7*A*). We also consider how these responses compare to the full-time (FT) stimulus (40–3,000 ms). Plotting the normalized temporal responses reveals that at the faster stimulus speeds, the initial transient response is predominant (Fig. 7*B*). At intermediate speeds (32–64°/s), the initial transient is also elevated, but the steady-state response is maintained. These trends are stronger for the preferred direction (green) but also present in the antipreferred direction (orange). At slow speeds (<4°/s), responses are minimal. The trends in temporal dynamics are particularly apparent by focusing on the first 500 ms of response for the four common speeds (Fig. 7*D*). The polar plots for all cellular responses are shown for each epoch of stimulus presentation (Fig. 7*C*). The overall population bias for TN motion at intermediate speeds is maintained throughout stimulus presentation. In contrast, the population bias for omnidirectional motion at faster speeds is strongest at the initial transient phase and reduced or absent thereafter.

**Figure 8.**
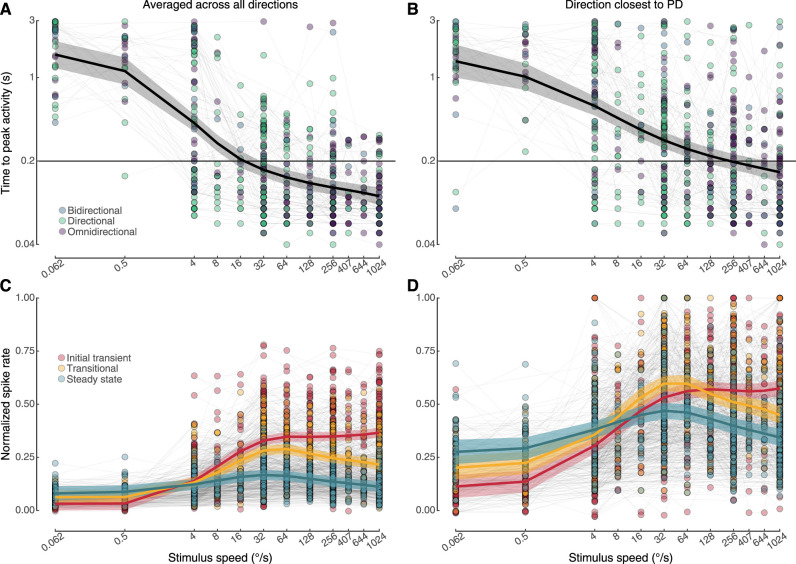
The temporal response sequence of LM neurons varies with direction preference and stimulus speed. The time to peak activity declines with stimulus speed both when averaged across all directions (***A***) and when analyzed only in the direction closest to the preferred direction (***B***). It takes longer, however, for the cells to reach peak activity when responding to the preferred direction. All axes are plotted on log scales. Each dot is a single cell's response at a given speed, and the lines connect the same cell tested at different speeds. The thick black curve (with 95% CI in gray) is the GAM model fit. The black horizontal line is the time that corresponds to the end of the initial transient phase. Under both analytical conditions (***C***, ***D***), the initial transient phase peaks at a higher stimulus speed than the transitional or steady-state responses. Steady-state responses are generally consistent across speeds whereas the initial transient and transitional phases show stronger speed dependence. Spike rates are normalized to the highest rate shown by each cell, in any direction, across the full motion epochs.

The analyses in Figure 7 indicate the temporal dynamics of the response to motion are important. We next asked how long it takes for the cells to reach peak activity following the onset of stimulus motion. This value is plotted for all cells at all speeds, either when averaged across all directions (Fig. 8*A*) or when only considered for the direction that was closest to the preferred direction (Fig. 8*B*). Each of best-fitting GAMs (Tables 3, 4) indicates that the time to peak normalized activity decreases monotonically as speed increases. These relationships were not affected by the shape of the tuning curve (directional, bidirectional, or omnidirectional). Time to peak activity does decline at a slower rate, however, when considering only the preferred direction.

**Table 3.** Goodness of fit metrics for models fit to explain log_2_ of time to peak response $(\mathrm{tim}e_{p})$

Formula	*R* ^2^	Root mean squared error	Sigma	AIC
$\log_{2}\;({\text{time}}_{p})∼\;(1\;|\;\text{cell})$	0.17	1.45	1.52	2,749.10
$\log_{2}\;({\text{time}}_{p})∼\;s(\log_{2}(\text{speed}))$	0.42	0.96	1.04	2,414.84
$\log_{2}\;({\text{time}}_{p})\;∼\;s(\log_{2}(\text{speed}))+(1\;|\;\text{cell})$	**0.42**	**0**.**96**	**1**.**04**	**2,308**.**35**
$\log_{2}\;({\text{time}}_{p})∼\;s(\log_{2}(\text{speed})\;*\;\text{shape})$	0.41	1.27	1.28	2,436.73
$\log_{2}\;({\text{time}}_{p})\;∼\;s(\log_{2}(\text{speed})\;*\;\text{shape})+(1\;|\;\text{cell})$	0.41	0.96	1.04	2,334.81

Data come from averaged responses across all directions for each cell, at each speed. Five GAM models were fit, with potential explanatory variables including log_2_ of stimulus speed (speed), shape category (shape; directional, omnidirectional, or bidirectional), and cell identity (cell; as a random effect). Sigma column denotes the residual standard deviation. The best-fitting model, determined by lowest AIC score, is in bold.

**Table 4.** Goodness of fit metrics for models fit to explain log_2_ of time to peak response $(\mathrm{tim}e_{p})$

Formula	R^2^	Root mean squared error	Sigma	AIC
$\log_{2}\;({\text{time}}_{p})∼\;(1\;|\;\text{cell})$	0.21	1.46	1.54	2,331.51
$\log_{2}\;({\text{time}}_{p})∼\;s(\log_{2}(\text{speed}))$	0.00	1.48	1.48	2,198.54
$\log_{2}\;({\text{time}}_{p})\;∼\;s(\log_{2}(\text{speed}))+(1\;|\;\text{cell})$	**0.26**	**1**.**20**	**1**.**28**	**2,136**.**02**
$\log_{2}\;({\text{time}}_{p})∼\;s(\log_{2}(\text{speed})\;*\;\text{shape})$	0.00	1.47	1.48	2,213.71
$\log_{2}\;({\text{time}}_{p})\;∼\;s(\log_{2}(\text{speed})\;*\;\text{shape})+(1\;|\;\text{cell})$	0.27	1.18	1.27	2,152.49

Data come from only the direction closest to the preferred direction for each cell, at each speed. Five GAM models were fit, with potential explanatory variables including log_2_ of stimulus speed (speed), shape category (shape; directional, omnidirectional, or bidirectional), and cell identity (cell; as a random effect). Sigma column denotes the residual standard deviation. The best-fitting model, determined by lowest AIC score, is in bold.

An earlier study of lobula plate tangential cells, specifically H1 cells, of the blowfly demonstrated that the transient response of the cells is biased for faster speeds than the steady response (Maddess and Laughlin, 1985). To determine if a similar phenomenon exists for zebra finch LM neurons, we examined the peak spike rate during the initial transient, transitional, and steady-state responses. The spike rates were normalized to the highest rate shown by each cell, in any direction, across the full motion epochs. When considering the responses averaged across all directions (Fig. 8*C*), the best-fitting GAM (Table 5) indicates that the steady responses were consistently low, with a slight peak at intermediate speeds (16–64°/s). The initial transient and transitional phases were more strongly biased for speed, with the peak of the transitional phases biased for intermediate speeds and the peak of the initial transient biased for faster speeds. When considering only the preferred direction (Fig. 8*D*), the best-fitting GAM (Table 6) indicated that overall responses were higher, but the transient response was still biased for faster speeds than either the steady-state or transitional responses.

**Table 5.** Goodness of fit metrics for models fit to explain the magnitude of peak activity within each phase (activity*_p_*)

Formula	R^2^	Root mean squared error	Sigma	AIC
$\text{activit}y_{p}\;∼\;(1\;|\;\text{cell})$	0.15	0.13	0.14	−2,419.55
$\text{activit}y_{p}\;∼\;\text{phase}+\;s(\log_{2}(\text{speed})\;*\;\text{phase})$	0.21	0.10	0.11	−3,465.61
$\text{activit}y_{p}\;∼\;\text{phase}+\;s(\log_{2}(\text{speed})\;*\;\text{phase})+\;(1\;|\;\text{cell})$	**0.41**	**0**.**09**	**0**.**94**	**−3,922**.**36**

Data come from averaged responses across all directions for each cell, at each speed. Three GAM models were fit, with potential explanatory variables including log_2_ of stimulus speed (speed), phase category (phase; initial transient, transitional, or steady state), and cell identity (cell; as a random effect). Sigma column denotes the residual standard deviation. The best-fitting model, determined by lowest AIC score, is in bold.

**Table 6.** Goodness of fit metrics for models fit to explain the magnitude of peak activity within each phase (activity*_p_*)

Formula	R^2^	Root mean squared error	Sigma	AIC
$\text{activit}y_{p}\;∼\;(1\;|\;\text{cell})$	0.16	0.20	0.21	−527.77
$\text{activit}y_{p}\;∼\;\text{phase}+\;s(\log_{2}(\text{speed})\;*\;\text{phase})$	0.03	0.19	0.19	−1,059.27
$\text{activit}y_{p}\;∼\;\text{phase}+\;s(\log_{2}(\text{speed})\;*\;\text{phase})+\;(1\;|\;\text{cell})$	**0.21**	**0**.**17**	**0**.**17**	**−1,317**.**23**

Data come from only the direction closest to the preferred direction for each cell, at each speed. Three GAM models were fit, with potential explanatory variables including log_2_ of stimulus speed (speed), phase category (phase; initial transient, transitional, or steady state), and cell identity (cell; as a random effect). Sigma column denotes the residual standard deviation. The best-fitting model, determined by lowest AIC score, is in bold.

## Discussion

We asked if the directional selectivity of midbrain neurons that respond to global visual motion changes across stimulus speeds. We made single unit recordings from the pretectal nucleus lentiformis mesencephali (LM) of zebra finches (*Taeniopygia guttata*) across a range of stimulus speeds by varying spatial and temporal frequency (Fig. 1). Cellular responses to stimulus direction could be characterized as directional, bidirectional, omnidirectional, or unmodulated using several metrics (Fig. 2). These metrics allowed for automated classification of cellular responses using machine learning (Fig. 3, Extended Data Figure 3-1). LM neurons were most responsive at intermediate stimulus speeds (32–64°/s; Fig. 4). Considering the responses across all speeds, the cells could be grouped into four general categories (Fig. 5): cells that (1) shifted from directionally selective at intermediate speeds to omnidirectionally responsive at faster speeds; (2) were directionally selective at most speeds; (3) were omnidirectionally responsive at most speeds; and (4) were variable in responses across speeds. As in our previous studies of zebra finch LM neurons (Gaede et al., 2017; Smyth et al., 2022), most of the cells were directional at 32°/s (*n* = 68 out of 114 cells), and the majority of those cells (*n* = 44) preferred temporal-to-nasal motion. We performed further analysis on how those responses in particular changed across speeds (Fig. 6). Only seven of the cells that were TN preferring at intermediate speeds remained directional at the fastest speed (1,024°/s). Of these cells, only three preferred TN motion at this speed. In contrast, many of the LM neurons were omnidirectionally responsive (*n* = 52 out of 114 cells) at the fastest speed. Thus, we observed an overall shift in the bias of LM neurons for temporal-to-nasal directional selectivity at intermediate speeds to omnidirectional responsiveness at very fast speeds. Lastly, we analyzed the temporal dynamics of the responses during stimulus motion, which revealed that the response had early onset and rapid offset at high stimulus speed (Figs. 7, 8). Overall, the measurements from LM neurons identify a previously uncharacterized shift in tuning such that at high speeds, the responses of many cells are rapid, transient, and omnidirectional.

Changes in the directional selectivity of pretectal neurons to global visual motion have also been reported in the wallaby NOT (Ibbotson and Mark, 1994). At slow speeds, wallaby NOT neurons preferred TN motion, but at high speeds, they were inhibited by motion in all directions. It was proposed that this inhibition was mediated by omnidirectional cells in or near the NOT. In contrast, we observed some of the same LM cells shifting from TN selective to omnidirectionally responsive across speeds. A comparison of these results suggests that population responses across speeds in the wallaby NOT and the zebra finch LM arise from different mechanisms.

Until very recently, the responses of neurons in the accessory optic system and pretectum to global visual motion from a diversity of animals were only tested at stimulus speeds up to 512°/s, and in most cases, the upper limit was closer to 100°/s. The resulting speed tuning curves have peak responses at values <100°/s. The first study of LM neurons in hummingbirds used random dot field stimuli that had a maximum stimulus speed of 80°/s (Gaede et al., 2017). This study was designed to test the hypothesis proposed by Iwaniuk and Wylie (2007) that the hypertrophied LM of hummingbirds would have a bias for slower speeds. In contrast, hummingbirds were found to have a bias for faster speeds although the values for the peak responses could not be identified for many cells as they were clearly above the upper limit for the stimulus. These results inspired us to shift from using dot field stimulus to sine-wave gratings that could be varied in spatial and temporal frequency (Smyth et al., 2022). Across the full spatiotemporal domain, this approach has an upper limit of 1,024°/s for the applied stimuli. Some cells from both zebra finches and Anna's hummingbirds (*Calypte anna*) were found to have peak responses above 100°/s. These responses, however, were only tested in the preferred direction due to the constraints of holding neurons across the full range of stimulus treatments to fully sample the spatiotemporal domain. The approach for the current study was to use a narrow set of spatial and temporal frequency stimulus combinations to maximize sampling across stimulus speeds but to test directional responses at each speed.

In the LM of pigeons and in the NOT of mammals, the cells can be divided into a slow and a fast population, often with a cutoff of 4°/s (Ibbotson and Price, 2001; Winship et al., 2006). Of the animals studied so far, hummingbirds and zebra finches are different in that LM neurons with peak responses at speeds <4°/s are rare. In the current data set, none of the zebra finch LM neurons had peak responses at slow speeds. Ibbotson and Price (2001) have argued that fast neurons are responsible for the initial phase of optokinetic nystagmus when the retinal slip velocity is high, and the slow neurons are responsible for driving optokinetic nystagmus when retinal slip velocities are low. It seems unlikely that zebra finches lack the ability to follow motion stimuli. As can be seen in Figure 8*D*, LM neurons in the zebra finch do respond to slow velocities (<4°/s), especially in the preferred direction albeit be a lower gain compared with the peak response. Thus, in zebra finches, responses to both slow and fast optokinetic nystagmus may be accomplished by some of the same cells, but with different temporal dynamics.

Global visual motion is also analyzed in other subcortical regions in vertebrates. The accessory optic system contains populations of neurons that prefer either upward or downward motion, and in some species, there is also a small population of NT-preferring cells (McKenna and Wallman, 1985; Soodak and Simpson, 1988; Simpson et al., 1988b; Wylie and Frost, 1990; Gaede et al., 2022). Both the pretectum and the accessory optic system send strong projections to the vestibulocerebellum, both through mossy fiber projections and climbing fiber projections through the inferior olive (Simpson, 1984; Wylie, 2000; Pakan et al., 2010). In mammals and in pigeons, the vestibulocerebellum is arranged into bands of selectivity for panoramic visual fields with different optic flow tuning (Graf et al., 1988; Kano et al., 1990; Kusunoki et al., 1990; Wylie et al., 1993). The general vertebrate pattern of anatomical connectivity has been confirmed in zebra finches (Gaede et al., 2019; Wylie et al., 2023). Because we are currently lacking measurements of neurons in the zebra finch vestibulocerebellum to global visual motion, it is unknown how these may be affected by speed-dependent changes in the directional selectivity of pretectal neurons.

Although LM is only one component of the midbrain–cerebellar pathway for optic flow processing, it is nonetheless worthwhile to consider what role it could have in flight control. In the zebra finch, the LM has a strong bias for temporal-to-nasal motion at intermediate stimulus speeds (Gaede et al., 2017; Smyth et al., 2022), whereas the nucleus of the basal optic root has a bias for upward and downward motion (Gaede et al., 2022). This division of direction preferences is generally consistent across vertebrates (Simpson et al., 1979; Burns and Wallman, 1981; Hoffmann and Schoppmann, 1981; Grasse and Cynader, 1984; Fite, 1985; McKenna and Wallman, 1985; Winterson and Brauth, 1985; Mustari and Fuchs, 1990; Rosenberg and Ariel, 1990; Ibbotson et al., 1994; Wylie and Crowder, 2000; Wylie, 2013). Given the bias of LM and its mammalian homolog for horizontal optic flow, and temporal-to-nasal motion in particular, why is there no major population of neurons in the midbrain for responses to nasal-to-temporal motion? It has been proposed that because this pathway is involved in stabilizing visual reflexes, it would be detrimental to have strong oculomotor responses to nasal-to-temporal motion given that this is the primary direction of optic flow during forward movement through the environment (Collewijn and Noorduin, 1972; Land, 2015). An alternative, nonexclusive hypothesis is that heightened sensitivity to temporal-to-nasal motion could be particularly beneficial in stabilizing whole-body locomotion by allowing animals to detect unwanted backward drift due to wind or water currents (Chapman et al., 2011). The only animal group documented so far that lacks an overall temporal-to-nasal bias is the pretectum in the hummingbird, which also is unique among vertebrates in its ability to sustain hovering flight (Gaede et al., 2017; Smyth et al., 2022). This result suggests to us that the direction and speed tuning in the midbrain–cerebellar optic flow pathways may have functional consequences for locomotor control in addition to their well-described role in eye stabilization.

Does the shift in bias from TN tuning to omnidirectional responses have a functional implication for zebra finch flight control? A distinct feature of optic signals is that optic flow velocity increases with proximity to a surface or edge in the environment (Gibson, 1954; Ibbotson, 2017). The population of LM neurons in the zebra finch is therefore expected to become very active as a bird flies very close to objects in its environment, even though this activity should have little if any directional signal. At very fast speeds, it may be challenging for the visual signal to encode direction accurately due to the temporal dynamics of local motion-detecting circuits and aliasing. It may also be that a proximity signal transmitted by the LM population does not need to have directional information to be useful for collision avoidance.

A well-known proximity signal in animal visual systems is the response to looming, especially to an expanding OFF stimulus (Klapoetke et al., 2017; Kim et al., 2020). Encoding of looming has been demonstrated in the tectofugal pathway of birds (Sun and Frost, 1998). We are not aware of any data suggesting that the accessory optic system and/or pretectum also contains looming-sensitive cells, but it may also be that looming stimuli have not been tested at sufficiently fast speeds to elicit such a response.

The hypothesis that LM neurons function as a warning system, signaling unexpected backward drift at intermediate optic flow velocity and signaling dangerous proximity at very fast optic flow velocity, could be tested during locomotion. If a flying zebra finch experiences temporal-to-nasal optic flow at intermediate speeds, it is expected to make a compensatory movement as it attempts to negate this regressive optic flow. It is predicted that this response will be abolished if the population of LM neurons that are TN preferring at intermediate speeds is inactivated pharmacologically or optogenetically. If a flying zebra finch experiences very fast optic flow, regardless of direction, it is expected to make a rapid avoidance response. Our data suggest that such a response would be driven by the initial transient response of the omnidirectionally sensitive LM neurons. It is therefore predicted that if this population of LM neurons could be briefly silenced during the first ∼200 ms of a fast omnidirectional stimulus presentation, any avoidance response should be eliminated or reduced. All of these predictions are based on the hypothesis that diverse response properties of the same LM neurons can be processed differently in downstream regions such as the cerebellum.

## Synthesis

Reviewing Editor: Fabienne Poulain, University of South Carolina Decisions are customarily a result of the Reviewing Editor and the peer reviewers coming together and discussing their recommendations until a consensus is reached. When revisions are invited, a fact-based synthesis statement explaining their decision and outlining what is needed to prepare a revision will be listed below. The following reviewer(s) agreed to reveal their identity: Michael R Ibbotson. Note: If this manuscript was transferred from JNeurosci and a decision was made to accept the manuscript without peer review, a brief statement to this effect will instead be what is listed below.

Thank you for submitting your manuscript to eNeuro. After careful consideration, the reviewers and I agree that your study analyzing the encoding of global visual motion in the avian pretectum is interesting and significant. Both reviewers also noted that your manuscript has high quality figures and analysis, is very clear and well written. However, reviewers also agree that your study would benefit from revising the discussion to better explain the interpretation of your results. Including a visual abstract would also be beneficial for readers who are not experts in the field. I am including each review below, so that you can address reviewers' comments in your resubmission.

Reviewer 1:

The manuscript of Dash et al., investigates whether the directional selectivity of neurons that respond to global visual motion changes across stimulus speeds. This question has been generally overlooked in the literature as the directional preference is usually tested at the neuron's preferred speed. By performing recordings in the nucleus LM of zebra finches and after a thorough characterization of the visual response properties of the neurons, the authors show that most neurons change their temporal to nasal directional preference at intermediate speeds to an omnidirectional preference at higher speeds. The changes in the responses' dynamics at different speeds are also described, and the overall possible functional significances of these findings are analyzed in the Discussion.

The manuscript is very clear and well written and the figures describe the results to the utmost details.

My only concern refers to the discussion, which I find too succinct and restrained, considering all the effort spent by the authors, and the future readers, in the detailed analysis of the results. There are many questions that in my opinion should be pondered, even though they might have been discussed in previous publications. This would make the manuscript more interesting to a wider audience.

For example, they report that most neurons are completely unresponsive below 4deg/s. Wouldn't that merit some comments, as the LM supposedly participates in stabilizing reflexes, like the optokinetic and optocollic reflexes, that operate in closed-loop, which require neurons responding to very slow speeds, presumably keeping their directional preference? In this respect calling the temporal to nasal optic flow as "aberrant" seems an overstatement, as eye and head instability may occur in all directions. The authors seem to imply that the LM is only involved in optic flow analysis to guiding correcting maneuvers during flight and forward movements, but this idea should be stated more clearly and be compared with alternative functions.

Also it is not explained in the text why an impending collision might produce a coherent temporal to nasal optic flow that could thus be signaled by the LM neurons. And what about the contribution of the LM neurons to translational and rotational optic flow responses in the cerebellum? How these responses could be affected by speed-dependent changes in the directional selectivity of input neurons?

In this regard, the second prediction stated at the end of the discussion is confusing. Does it imply that as the LM neurons shift their preference for different speeds, the "corrective maneuvers" that they contribute to generate would change accordingly? Would these maneuvers be compensatory throughout the entire velocity range?

Minor issues.

The abstract should highlight some of the possible behavioral implications of the results.

In spite of the word limits, it would be better if the sentence on line 37 started, "We found that..."

The pretectum is a major anatomical division containing several nuclei not all responding to optic flow; the first paragraph of the introduction seems to imply that.

In that respect, Line 52 should read, "neurons from these regions ...." instead of "these neurons

Figure Legends

Figure 1. What is the purpose of showing an arrow in the stationary, gray part of the stimulus?

Figure 2. The raster representation is very confusing, as the continuum of black spikes looks as the background and the thin green lines look as spikes. I recommend drawing a classical raster display with black dots as spikes on a white background or at least state clearly in the legend that spikes are represented in black.

There seems to be a second Figure 3 at the end of the list of figures, with more elements than the ones described in the text and figure legend of the first one.

Reviewer 2:

This paper has high quality figures and analysis. The analysis is highly quantitative and reveals a set of complex and rigorous results that are of great interest. I think the discussion doesn't capture everything that the data shows and some extra analysis might be quite revealing. I found Figure 5H and 5I incredibly difficult to understand (i.e. the Sankey plots). There must be a simpler way.

I will focus quite heavily on Figure 5c as this is the population summary. I note that there may be several cell classes if a holistic approach is taken, rather than trying to define classes via shifts in tuning.

1. In the bottom right corner of 5c we find about 36 neurons that are directional at 32degs/s and below. Moderate responses persist at higher speeds but they become omni-directional. These cells are very interesting and rarely encountered in other species. What is the mechanism and function?

2. Moving upwards, we find a block of about 32 cells that are mainly directional at/below 32degs/s and variable above. Some are omni, some directional and some unresponsive. These look very like the DS cells reported in wallaby NOT. I interpret this as: DS breaks down at high speeds leading to increased variability in responses.

3. Above them is a block of about 30 cells that are omnidirectional at most speeds, with some being either directional or unresponsive at low and high speeds. Some of these resemble the non-directional, high-speed sensitive neurons in wallaby NOT.

4. Finally, at the top there are 10-15 cells that have mixed responses, either unresponsive or bidirectional. Not clear what these are. Maybe they do not like grating stimuli.

Maybe I am making too much of this. However, if the cells were clustered quantitatively, guided roughly by my comments above, there might be evidence for a few cell classes. If so, does this result suggest different mechanisms and different functions? The paper makes one main conclusion: that omnidirectional responses at high speeds might offer an ability to generate avoidance responses. This is a functional explanation. A more general result might be that the system needs a range of cell types to do LM's overall job.

On the question of a mechanism for the omnidirectional responses, for group 3 above, it could be as simple as the cells being wide-field brightness detectors, perhaps to calibrate the system when a bird flies from a dark thicket into the open sky. If so, they might resemble the wide-field Y-type cells in mammalian retina, which have been speculated by some as calibration cells. Maybe LM needs transient signals of this type to prevent the system going out of control when the DS cells are overwhelmed by a sudden brightness change (DS cells are often transiently responsive to flashed stimuli). Did the authors test the cells with non-motion stimuli, such as changes in brightness? Maybe omni-directional cells are simply brightness transition detectors, for want of a better term? If so, this is a significant finding for LM, usually considered to be only a motion processing area.

I am very pleased to see that the authors looked at the time scale of the responses. Clearly, the temporal response sequence is critically important. The authors note that high-speed responses are transient, while lower speed responses are ongoing. This was noted way back in 1985 by Ted Maddess in fly neurons. He showed that peak speed sensitivity is biased towards high speeds for the transient response but for lower speeds for the ongoing response. Indeed, Figure 6c resembles the critical plot in Maddess's paper. Some discussion of why this might be important is merited. I presume that LM helps to stabilise the head and body against continuous small changes, each of which would generate transient shifts in the image. Is it the transient response that matters most during real behaviour? What might this finding say about mechanisms?

Overall, I wonder if simplifying the classification story might be a positive way forward. Again, the classification outlined in Figure 5 HI is challenging! A discussion of mechanisms might help distinguish between the characteristics that are functionally useful and those that are simply a side-effect of operating over such huge speeds ranges. At high speeds, blur creates visual streaks and this can change the way DS can be calculated. I'd like to see this pursued in the discussion.

## Author Response

Synthesis of Reviews:

Thank you for submitting your manuscript to eNeuro. After careful consideration, the reviewers and I agree that your study analyzing the encoding of global visual motion in the avian pretectum is interesting and significant. Both reviewers also noted that your manuscript has high quality figures and analysis, is very clear and well written. However, reviewers also agree that your study would benefit from revising the discussion to better explain the interpretation of your results. Including a visual abstract would also be beneficial for readers who are not experts in the field. I am including each review below, so you can address reviewers' comments in your resubmission.

Thank you for the positive and very helpful assessment. Both reviewers provided excellent guidance for us to revise our discussion to clarify and broaden our interpretation of the results. Point-by-point responses are provided below.

Reviewer 1:

The manuscript of Dash et al., investigates whether the directional selectivity of neurons that respond to global visual motion changes across stimulus speeds. This question has been generally overlooked in the literature as the directional preference is usually tested at the neuron's preferred speed. By performing recordings in the nucleus LM of zebra finches and after a thorough characterization of the visual response properties of the neurons, the authors show that most neurons change their temporal to nasal directional preference at intermediate speeds to an omnidirectional preference at higher speeds. The changes in the responses' dynamics at different speeds are also described, and the overall possible functional significances of these findings are analyzed in the Discussion.

The manuscript is very clear and well written and the figures describe the results to the utmost details.

We are so pleased that the manuscript was deemed as interesting and clear.

My only concern refers to the discussion, which I find too succinct and restrained, considering all the effort spent by the authors, and the future readers, in the detailed analysis of the results. There are many questions that in my opinion should be pondered, even though they might have been discussed in previous publications. This would make the manuscript more interesting to a wider audience.

The second reviewer shared this concern, and both reviewers offered excellent advice for expanding the discussion. We have added multiple paragraphs as described below.

For example, they report that most neurons are completely unresponsive below 4deg/s. Wouldn't that merit some comments, as the LM supposedly participates in stabilizing reflexes, like the optokinetic and optocollic reflexes, that operate in closed-loop, which require neurons responding to very slow speeds, presumably keeping their directional preference? In this respect calling the temporal to nasal optic flow as "aberrant" seems an overstatement, as eye and head instability may occur in all directions. The authors seem to imply that the LM is only involved in optic flow analysis to guiding correcting maneuvers during flight and forward movements, but this idea should be stated more clearly and be compared with alternative functions.

Good point. We have added the following paragraph to the discussion: "In the LM of pigeons and in the NOT of mammals, the cells can be divided into a slow and a fast population, often with the cutoff of 4{degree sign}/s (Ibbotson and Price, 2001; Winship et al., 2006). Of the animals studied so far, hummingbirds and zebra finches are different in that LM neurons with peak responses at speeds < 4{degree sign}/s are rare. In the current data set, none of the zebra finch LM neurons had peak responses at slow speeds. Ibbotson and Price (Ibbotson and Price, 2001) have argued that fast neurons would be responsible for the initial phase of optokinetic nystagmus when the retinal slip velocity is high, and the slow neurons are responsible for driving optokinetic nystagmus when retinal slip velocities are low. It seems unlikely that zebra finches lack the ability to follow motion stimuli. As can be seen in figure 8D, LM neurons in the zebra finch do respond to slow velocities (<4{degree sign}/s), especially in the preferred direction albeit be a lower gain compared to the peak response. Thus, in zebra finches, responses to both slow and fast OKN may be accomplished by some of the same cells, but with different temporal dynamics." Also it is not explained in the text why an impending collision might produce a coherent temporal to nasal optic flow that could thus be signaled by the LM neurons.

Thank you for pointing this out to us. We thought a lot about this comment, and made extensive changes to the end of the discussion to clarify our arguments about potential significance for locomotor control. We addressed this concern over the last four paragraphs of the new version of the discussion.

And what about the contribution of the LM neurons to translational and rotational optic flow responses in the cerebellum? How these responses could be affected by speed-dependent changes in the directional selectivity of input neurons? Thanks for pointing this out to us. We have added the following paragraph to the discussion to consider these questions: "Global visual motion is also analyzed in other subcortical regions in vertebrates. The accessory optic system contains populations of neurons that prefer either upward or downward motion, and in some species, there is also a small population of NT preferring cells (McKenna and Wallman, 1985; Simpson et al., 1988b; Soodak and Simpson, 1988; Wylie and Frost, 1990; Gaede et al., 2022). Both the pretectum and the accessory optic system sends strong projections to the vestibulocerebellum, both through mossy fibre projections and climbing fibre projections through the inferior olive (Simpson, 1984; Wylie, 2000; Pakan et al., 2010). In mammals and in pigeons, the vestibulocerebellum is arranged into bands of selectivity for panoramic visual fields with different optic flow tuning (Graf et al., 1988; Kano et al., 1990; Kusunoki et al., 1990; Wylie et al., 1993). The general vertebrate pattern of anatomical connectivity has been confirmed in zebra finches (Gaede et al., 2019; Wylie et al., 2023). Because we are currently lacking measurements of neurons in the zebra finch vestibulocerebellum to global visual motion, it is unknown how these may be affected by speed-dependent changes in the directional selectivity of pretectal neurons." In this regard, the second prediction stated at the end of the discussion is confusing. Does it imply that as the LM neurons shift their preference for different speeds, the "corrective maneuvers" that they contribute to generate would change accordingly? Would these maneuvers be compensatory throughout the entire velocity range? We apologize for the confusion. We have rewritten this section, which now reads, "One prediction is an animal will make a compensatory movement as it attempts to negate regressive optic flow, but that the behavior will be abolished when LM is inactivated pharmacologically or optogenetically. A second prediction is that compensatory movements should occur when an animal is exposed to regressive optic flow at intermediate speeds, but that fast stimuli should elicit a rapid avoidance response regardless of the direction of optic flow." Minor issues.

The abstract should highlight some of the possible behavioral implications of the results.

Thank you for the great suggestion. We now conclude the abstract with the following sentence: "These results suggest a potential role for the pretectum during flight by detecting unexpected drift or potentials collisions, depending on the speed of the optic flow signal." In spite of the word limits, it would be better if the sentence on line 37 started, "We found that..." Changed as suggested The pretectum is a major anatomical division containing several nuclei not all responding to optic flow; the first paragraph of the introduction seems to imply that.

In that respect, Line 52 should read, "neurons from these regions ...." instead of "these neurons Changed as suggested Figure Legends Figure 1. What is the purpose of showing an arrow in the stationary, gray part of the stimulus? Good point. We have added the following text to the figure legend for 1B: "Arrows indicate the orientation of the stimulus (grey), and both orientation and direction (green)." Figure 2. The raster representation is very confusing, as the continuum of black spikes looks as the background and the thin green lines look as spikes. I recommend drawing a classical raster display with black dots as spikes on a white background or at least state clearly in the legend that spikes are represented in black.

We have addressed this concern in two ways. First, we reduced the height of the spike lines so that the timing relative to stimulus presentation is easier to see. Second, we followed your specific suggestion and added the following text to the legend for panel 2B: "Black vertical lines indicate individual spike timing." There seems to be a second Figure 3 at the end of the list of figures, with more elements than the ones described in the text and figure legend of the first one.

Our apologies for the confusion. The second version is an extended version. We neglected to include the legend for figure 3 extended. This has now been added to manuscript.

Reviewer 2:

This paper has high quality figures and analysis. The analysis is highly quantitative and reveals a set of complex and rigorous results that are of great interest. I think the discussion doesn't capture everything that the data shows and some extra analysis might be quite revealing. I found Figure 5H and 5I incredibly difficult to understand (i.e. the Sankey plots). There must be a simpler way.

We see your points. We have extensively to the discussion based on both reviewers' feedback. We addressed the specific concern about figure 5H and 5I by moving these to a new figure (figure 6), and by incorporating your next set of suggestions in new panels in figure 5 (H-K).

I will focus quite heavily on Figure 5c as this is the population summary. I note that there may be several cell classes if a holistic approach is taken, rather than trying to define classes via shifts in tuning.

1. In the bottom right corner of 5c we find about 36 neurons that are directional at 32degs/s and below. Moderate responses persist at higher speeds but they become omni-directional. These cells are very interesting and rarely encountered in other species. What is the mechanism and function? 2. Moving upwards, we find a block of about 32 cells that are mainly directional at/below 32degs/s and variable above. Some are omni, some directional and some unresponsive. These look very like the DS cells reported in wallaby NOT. I interpret this as: DS breaks down at high speeds leading to increased variability in responses.

3. Above them is a block of about 30 cells that are omnidirectional at most speeds, with some being either directional or unresponsive at low and high speeds. Some of these resemble the non-directional, high-speed sensitive neurons in wallaby NOT.

4. Finally, at the top there are 10-15 cells that have mixed responses, either unresponsive or bidirectional. Not clear what these are. Maybe they do not like grating stimuli.

Maybe I am making too much of this. However, if the cells were clustered quantitatively, guided roughly by my comments above, there might be evidence for a few cell classes. If so, does this result suggest different mechanisms and different functions? The paper makes one main conclusion: that omnidirectional responses at high speeds might offer an ability to generate avoidance responses. This is a functional explanation. A more general result might be that the system needs a range of cell types to do LM's overall job.

Thanks for these ideas. We have incorporated your idea that the cells could be grouped into these four categories. The different groups are now displayed in figure 5H-K, and discussed in both the results and the discussion. We decided to keep the original versions of figure 5H,I in the new figure 6 so that the connections among responses at different speeds can be seen.

On the question of a mechanism for the omnidirectional responses, for group 3 above, it could be as simple as the cells being wide-field brightness detectors, perhaps to calibrate the system when a bird flies from a dark thicket into the open sky. If so, they might resemble the wide-field Y-type cells in mammalian retina, which have been speculated by some as calibration cells. Maybe LM needs transient signals of this type to prevent the system going out of control when the DS cells are overwhelmed by a sudden brightness change (DS cells are often transiently responsive to flashed stimuli). Did the authors test the cells with non-motion stimuli, such as changes in brightness? Maybe omni-directional cells are simply brightness transition detectors, for want of a better term? If so, this is a significant finding for LM, usually considered to be only a motion processing area.

This is an interesting idea. We were able to explore it by comparing the responses to the blank (white) screen with responses to the stationary stimulus pattern (see white and gray periods for the representative cells in figure 2A,B,C). In other words, there is a built-in reduction in luminance for each recording sweep. This analysis did not show any overall change in modulation with reduction in luminance for any of the cell classifications. Thus, there does not seem to be any evidence that any of the cells are functioning as brightness transition detectors. We decided not to include this analysis as a supplementary figure, because there are some additional complications related to sample size and different spatial frequencies (see figure 1C, x-axis). We did see some evidence for a mild increase in response for transitions between the white screen and the highest spatial frequency patterns. It would be difficult to make a convincing analysis of that phenomenon, and in any case did not differ for omnidirectional versus other cell classifications. Based on these results, we felt it would be best to keep this in mind for a potential future study. We hope that is acceptable.

I am very pleased to see that the authors looked at the time scale of the responses. Clearly, the temporal response sequence is critically important. The authors note that high-speed responses are transient, while lower speed responses are ongoing. This was noted way back in 1985 by Ted Maddess in fly neurons. He showed that peak speed sensitivity is biased towards high speeds for the transient response but for lower speeds for the ongoing response. Indeed, Figure 6c resembles the critical plot in Maddess's paper. Some discussion of why this might be important is merited. I presume that LM helps to stabilise the head and body against continuous small changes, each of which would generate transient shifts in the image. Is it the transient response that matters most during real behaviour? What might this finding say about mechanisms? Thanks for the great suggestion. We decided to add a similar analysis to Maddess and Laughlin's (1985) figure 13. Our version is now shown in figure 8C,D. We are also seeing that the transient responses peak at higher speeds than the steady-state (and transitional) responses. We added the following paragraph to the results: "An earlier study of lobula plate tangential cells, specifically H1 cells, of the blowfly demonstrated that the transient response of the cells is biased for faster speeds than the steady response (Maddess and Laughlin, 1985). To determine if a similar phenomenon exists for zebra finch LM neurons, we examined the peak spike rate during the initial transient, transitional, and steady-state responses. The spike rates were normalized to the highest rate shown by each cell, in any direction, across the full motion epochs. When considering the responses averaged across all directions (Figure 8C), the best fitting GAM (Table 4) indicates that the steady responses were consistently low, with a slight peak at intermediate speeds (16-64{degree sign}/s). The initial transient and transitional phases were more strongly biased for speed, with the peak of the transitional phases biased for intermediate speeds, and the peak of the initial transient biased for faster speeds. When considering only the preferred direction (Figure 8D), the best fitting GAM (Table 5) indicated that overall responses were higher, but the transient response was still biased for faster speeds than either the steady-state or transitional responses." Overall, I wonder if simplifying the classification story might be a positive way forward. Again, the classification outlined in Figure 5 HI is challenging! A discussion of mechanisms might help distinguish between the characteristics that are functionally useful and those that are simply a side-effect of operating over such huge speeds ranges. At high speeds, blur creates visual streaks and this can change the way DS can be calculated. I'd like to see this pursued in the discussion.

Thank you for these suggestions. We agree that the categories you suggested provided a better framework for presenting and discussing the results. We hope the new version of figure 5(H-K) and figure 8 (C,D) improved the discussion along the lines you had in mind.

We also explored the idea of incorporating visual streaking due to motion blur as a mechanism for direction encoding. We went through the literature, including key papers by Wilson and Wilkinson, 1998 (Vision Research), Geisler (1999, Nature), Ross et al., 2000 (Current Biology), and Barlow and Olshausen, 2004 (Journal of Vision). Our take is that the proposed mechanism is based on orientation-selective cells, such as in mammalian V1 or in primate V4. We have not yet found any evidence of orientation selectivity in LM cells. We also considered a version of streak line encoding via the bi-directional cells that we describe. The low number of cells and the fact that they tended to encode slower speeds, but not consistently across speeds, made it difficult to consider them in this context. In summary, we were unable to come up with some text that we felt added much to the previous work on this topic. We would be happy to consider this further if the referee feels that we have missed the key connection.
