# Supplementary figures and images for: Encoding of Global Visual Motion in the Avian Pretectum Shifts from a Bias for Temporal-to-Nasal Selectivity to Omnidirectional Excitation across Speeds
Source: eNeuro. 2024 Dec 12;11(12):ENEURO.0301-24.2024. doi: 10.1523/ENEURO.0301-24.2024 (PMC11675535; doi:10.1523/ENEURO.0301-24.2024)

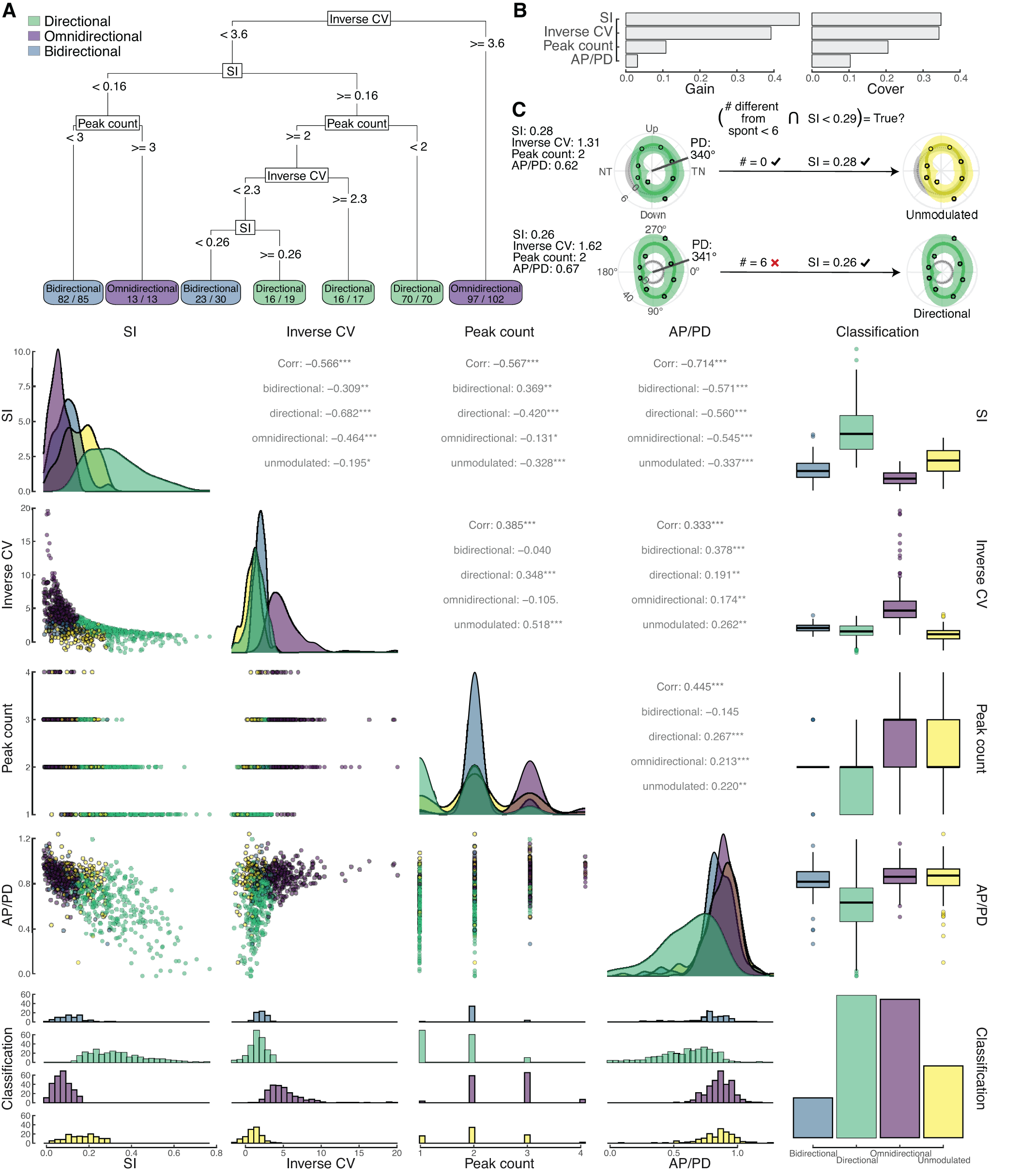

Supplement: Figure 3-1 — Pretectal neurons were classified in two stages using several measures of neural activity. In the first stage, cells were classified as directional, bidirectional, or omnidirectional based on selectivity index (SI), inverse of the coefficient of variation (CV), ratio of firing rate in the anti-preferred to preferred direction (AP/PD), and peak count. A) A representative example of a decision tree used by XGBoost to classify cells in the first stage is shown. This example has high accuracy for the training data for which it was supplied, based on the success ratios shown at the bottom. The XGBoost model was built from > 2500 decision trees. B) The relative contribution (gain) and relative number of observations (cover) in the consensus model reveals that SI and inverse CV were the most informative, whereas peak count and AP/PD provide refinement. C) In the second stage, cells can be reclassified as unmodulated if two conditions were true: i) fewer than six directions had mean firing rates that were significantly different from the spontaneous rate, and ii) SI ≤ 0.29. This step is illustrated for two cells with similar preferred directions (PD) and similar activity characteristics. The upper cell is reclassified as unmodulated because its activity in most directions is indistinguishable from spontaneous firing rate (grey circle) and its SI = 0.29. The lower cell is directional even though its SI is lower because its activity in six directions is above the spontaneous rate. It is not bidirectional because its SI is > ~0.2. Mean spontaneous rate has been subtracted from all data and is therefore shown at 0 spikes/s (grey). D) A matrix of plots for SI, Inverse CV, Peak count, AP/PD, and classification, colored by classification. Each row and column is labeled with one of the five variables. The plots on the unity diagonal show density histograms. Cells below the diagonal are bivariate plots. Cells above the diagonal provide overall correlations and classification-specifi [file eneuro-11-ENEURO.0301-24.2024-s001.tif]
